# Advantage of grading classification using volumetric artificial intelligence for periventricular hyperintensity and deep subcortical white matter hyperintensity

**DOI:** 10.1038/s41598-025-23859-2

**Published:** 2025-11-17

**Authors:** Masashi Kuwabara, Fusao Ikawa, Shinji Nakazawa, Saori Koshino, Daizo Ishii, Hiroshi Kondo, Takeshi Hara, Shingo Matsuda, Yuyo Maeda, Shiyuki Maeyama, Yoshinobu Seo, Jinichi Sasanuma, Kimito Kondo, Nobutaka Horie

**Affiliations:** 1 Department of Neurosurgery, Graduate School of Biomedical and Health Sciences, Hiroshima University, 1-2-3 Kasumi, Minami-ku, Hiroshima, Hiroshima, 734-8551 Japan; 2 Department of Neurosurgery, Shimane Prefectural Central Hospital, 4-1-1 Himebara, Izumo, Shimane, 693- 8555 Japan; 3 LPIXEL Inc, 1-6-1 Otemachi, Chiyoda-ku, Tokyo, 100-0004 Japan; 4 Department of Radiology, The University of Tokyo Hospital, 7-3-1 Hongo, Bunkyo-ku, Tokyo, 113-8655 Japan; 5 Department of Neurosurgery, Nakamura Memorial Hospital, South-1, West-14, Chuo-ku, Sapporo, 060-8570 Hokkaido Japan; 6 Department of Neurosurgery, Shin-yurigaoka General Hospital, 255, Furusawa-Miyako, Kawasaki Asao-ku, Kanagawa, 215-0026 Japan; 7 Department of Neurology, Hokuto Hospital, 7-5 Kisen, Inada-cho, Obihiro, 080- 0833 Hokkaido Japan

**Keywords:** Periventricular hyperintensity, Deep and subcortical white matter hyperintensity, Artificial intelligence, Grading, Magnetic resonance imaging, Software, Neurology, White matter disease

## Abstract

We developed and validated an artificial intelligence (AI) algorithm for the automated grading of periventricular hyperintensity (PVH) and deep subcortical white matter hyperintensity (DWMH) using magnetic resonance imaging. Overall, 246 patients were evaluated, with 137 and 109 allocated to the training and testing groups, respectively. AI-predicted grading according to the Fazekas scale was compared with expert assessments using accuracy, F1-score, and mean absolute error. Inter-rater agreement was evaluated using Fleiss’ kappa to assess consistency among human raters and Cohen’s kappa to measure agreement between the AI and individual human raters. The AI demonstrated superior multi-class accuracy in PVH classification compared with the human expert, achieving an accuracy of 0.798 versus 0.743. In DWMH classification, the AI outperformed the expert specifically in distinguishing Fazekas 0/1/2 from the 3 classification, achieving an accuracy of 0.954 compared with the expert’s 0.927. Inter-rater agreement analysis showed that for PVH and DWMH, the AI achieved “good agreement” with human raters. For PVH, the AI’s agreement exceeded the human inter-rater agreement. The developed AI also exhibited lower variability in volume ratio distribution within the same grade compared with human raters. The developed AI algorithm effectively distinguished between PVH and DWMH, achieving accuracy comparable to human performance.

**Supplementary Information:**

The online version contains supplementary material available at 10.1038/s41598-025-23859-2.

## Introduction

White matter hyperintensity (WMH) lesions reflect chronic hypoperfusion of the cerebral white matter, becoming more prevalent with age and being associated with cognitive dysfunction, ischemic cerebrovascular disease, affective disorders, and depression, depending on lesion severity^1–9^. Early detection and prevention of WMHs through routine brain examinations are considered essential^10,11^. Severe periventricular hyperintensity (PVH) and deep subcortical white matter hyperintensity (DWMH) are independent risk factors for stroke, with odds ratios of 4.7 and 3.6, respectively^12^. Furthermore, individuals with severe WMH have a threefold higher adjusted risk of stroke compared with those with minimal WMH^13^. Data from Japanese brain health screening programs (Brain Dock) have similarly indicated that severe PVH and advanced WMH serve as significant predictors of stroke, with severe PVH also being associated with increased mortality risk^14^. Meta-analyses have confirmed that moderate-to-severe WMH approximately doubles to triples the risk of stroke and death^15^, highlighting the need for early detection and intervention in cerebral small vessel disease.

Japan introduced the Brain Dock system—a comprehensive brain checkup program—in 1988 to facilitate the diagnosis of brain-related diseases and identification of early-stage abnormalities^1,16–18^. Traditionally, WMH grading has relied on manual interpretation based on physicians’ subjective judgment^19^. However, this approach is time-consuming, physically demanding, and subject to substantial interobserver variability, with reported rates ranging from 10% to 68%^19–21^. Recent studies have explored the automatic measurement of WMH volume using machine learning algorithms, with convolutional neural networks predominantly employed for WMH segmentation^2,21–25^. However, only a few studies have investigated algorithms capable of performing WMH segmentation using fluid-attenuated inversion recovery (FLAIR) images alone while quantitatively distinguishing PVH from DWMH through quantitative grading^19,21,26,27^. The Fazekas scale, widely applied in magnetic resonance imaging (MRI) assessments, remains the most commonly used method for assessing WMHs and differentiating PVH from DWMH^28–30^.

Distinguishing between PVH and DWMH is clinically important due to their differing pathological characteristics. Pathologically, PVH primarily reflects non-vascular changes, whereas DWMH is more closely associated with vascular pathology^31–33^. PVH mainly results from the destruction of the ependymal lining of the lateral ventricles and subependymal gliosis, processes not primarily vascular in origin. By contrast, DWMH is predominantly attributed to the dilatation of perivascular myelin caused by hypoxia resulting from atherosclerotic changes and the enlargement of the perivascular space due to fiber loss, reflecting a vascular pathology^31–33^. Additional studies have suggested that PVH results from impaired cerebrospinal fluid clearance due to glymphatic pathway dysfunction, whereas DWMH is attributed to chronic ischemic hypoperfusion in combination with glymphatic dysfunction^34^. From a clinical perspective, although both PVH and DWMH are associated with cognitive impairment, PVH has been reported to exert a greater impact on processing speed and executive function compared with DWMH. From a genetic perspective, a genome-wide association study of PVH and DWMH in 26,654 participants aged ≥ 45 years identified distinct genetic structures between the two^35^. Collectively, these findings underscore the importance of distinguishing PVH from DWMH on MRI, given their differing pathological, clinical, and genetic profiles.

Clinical applications of artificial intelligence (AI) for WMH grading have demonstrated consistently accurate diagnostic performance. However, a few multicenter studies have validated AI algorithms for automated grading^27,36,37^. This study aimed to develop and validate a novel AI grading system for PVH and DWMH using FLAIR imaging alone and highlight the limitations of qualitative classification, even among expert physicians. To achieve this, a practical pipeline was designed that integrates WMH segmentation, PVH/DWMH separation, and data-driven grading based on learned volume thresholds. Unlike previous approaches, which either omitted the separation step or relied on computationally intensive per-slice distance map calculations, the present method employs a morphology-based approximation using a ventricular mask^21,38^. This approach enables more efficient processing, rendering it suitable for clinical use.

## Results

### Grading annotations, WMH segmentation, and PVH and DWMH separation

**Fig. 1:**
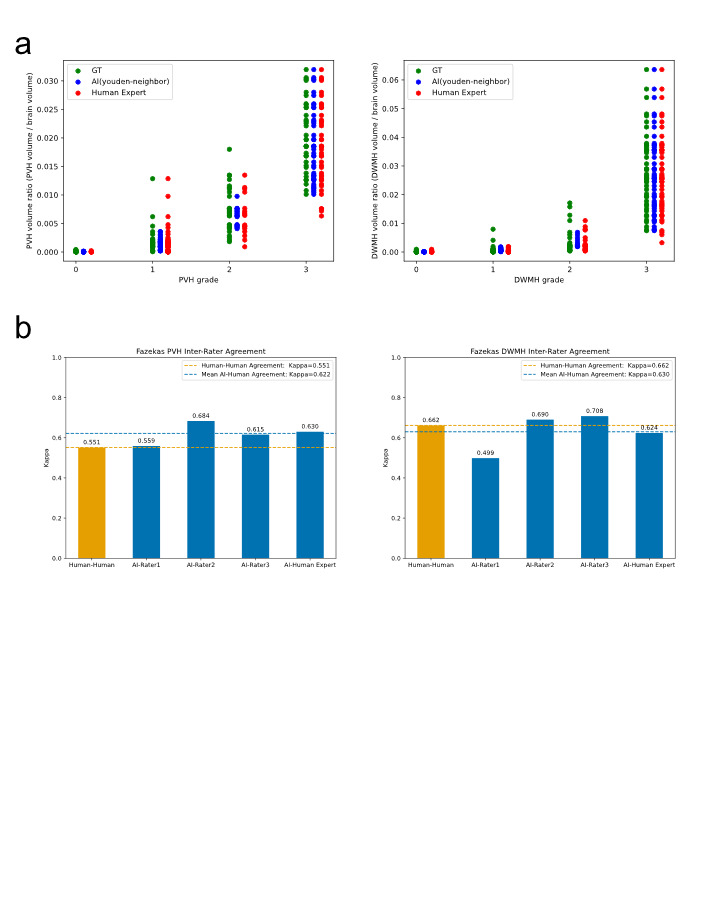
(**a**) Distribution of grades and AI-predicted volume ratios for Fazekas PVH and DWMH. The horizontal axis represents three grading sources: ground truth (GT), AI using the Youden-neighbor method, and human expert). The vertical axis indicates the AI-predicted volume ratios. Data were derived from the test dataset. The left plot displays the PVH grade distribution, whereas the right plot shows the DWMH grade distribution. (**b**) Inter-rater agreement for Fazekas PVH and DWMH. The yellow bar represents the agreement between human raters, calculated using Fleiss’ kappa based on the grading of four raters: rater 1, rater 2, rater 3, and the human expert. The blue bars indicate the agreement between the AI and each human rater, calculated using Cohen’s kappa. The blue dashed line shows the mean Cohen’s kappa across all AI-human pairs. All evaluations were conducted on the test dataset. The left panel illustrates PVH inter-rater agreement, whereas the right panel shows DWMH inter-rater agreement.

Table 1 presents the annotation results, detailing the number of patients in each grade and the mean ± standard deviation of PVH and DWMH volumes as quantified by the proposed method. A positive correlation was observed between severity grade and total WMH volume across all patients. Figure 1a illustrates the distribution of PVH and DWMH volume ratios corresponding to each Fazekas grade in the test dataset, with correlation coefficients of *r* = 0.840 for PVH and *r* = 0.729 for DWMH.

**Table 1:** Results of annotated WMH grading scales.

Training dataset (*n* = 137)		PVH		DWMH		Test dataset (*n* = 109)		PVH		DWMH	
Fazekas	Brain Dock	Patients	PVH volume mean (std) [mL]	Cases	DWMH volume mean (std) [mL]	Fazekas	Brain Dock	Patients	PVH volume mean (std) [mL]	Cases	DWMH volume mean (std) [mL]
0	0	32	0.451 (1.442)	30	0.451 (1.330)	0	0	18	0.087 (0.134)	15	0.158 (0.310)
1	1	46	1.535 (1.322)	40	0.790 (0.845)	1	1	29	1.966 (2.489)	23	0.910 (1.824)
2	2	23	8.456 (5.443)	31	4.816 (5.420)	2	2	28	6.996 (3.930)	25	3.849 (4.926)
3	3	20	13.503 (5.642)	24	20.353 (13.954)	3	3	25	17.515 (4.993)	32	21.397 (9.491)
	4	16	18.632 (10.520)	12	31.411 (13.839)		4	9	20.220 (6.486)	14	35.085 (11.635)

DWMH Deep and subcortical white matter hyperintensity, PVH Periventricular hyperintensity, std Standard deviation, WMH White matter hyperintensity.

### Quantitative comparison of grading

Table 2 summarizes the comparison of grading classification accuracy for the test dataset. Among the three thresholding methods, the “Youden-neighbor” method demonstrated superior performance, achieving the highest multi-class accuracy for Fazekas DWMH and Brain Dock PVH and DWMH classifications and ranking second for Fazekas PVH (Supplementary Fig. S1).

**Table 2:** Grading performance metrics for the Fazekas and brain dock scales.

		Accuracy					F1-score					MAE
		0vs123	01vs23	012vs3	Multi-class		0vs123	01vs23	012vs3	Multi-class		
Fazekas PVH	AI (density)	0.963	0.927	0.917	0.817		0.965	0.927	0.920	0.804		0.193
	AI (Youden-all)	0.954	0.927	0.844	0.734		0.956	0.927	0.850	0.696		0.275
	AI (Youden -neighbor)	0.945	0.927	0.917	0.798		0.944	0.927	0.920	0.789		0.211
	Human Expert	0.899	0.917	0.927	0.743		0.880	0.918	0.928	0.724		0.257
Fazekas DWMH	AI (density)	0.881	0.872	0.927	0.679		0.890	0.874	0.926	0.678		0.321
	AI (Youden-all)	0.817	0.872	0.945	0.688		0.841	0.874	0.945	0.661		0.367
	AI (Youden-neighbor)	0.908	0.872	0.954	0.743		0.913	0.874	0.954	0.731		0.266
	Human Expert	0.954	0.908	0.927	0.789		0.952	0.909	0.927	0.783		0.211
												MAE
		0vs1234	01vs234	012vs34	0123vs4	multi-class	0vs1234	01vs234	012vs34	0123vs4	multi-class	
Brain Dock PVH	AI (density)	0.963	0.927	0.908	0.789	0.606	0.965	0.927	0.911	0.832	0.608	0.413
	AI (Youden-all)	0.954	0.927	0.844	0.615	0.505	0.956	0.927	0.850	0.698	0.471	0.661
	AI (Youden-neighbor)	0.945	0.927	0.917	0.908	0.706	0.944	0.927	0.920	0.912	0.707	0.303
	Human expert	0.899	0.917	0.927	0.881	0.633	0.880	0.918	0.928	0.900	0.627	0.376
Brain Dock DWMH	AI (density)	0.881	0.872	0.936	0.826	0.514	0.890	0.874	0.936	0.850	0.499	0.486
	AI (Youden-all)	0.817	0.872	0.945	0.752	0.459	0.841	0.874	0.945	0.793	0.426	0.615
	AI (Youden-neighbor)	0.908	0.872	0.945	0.844	0.578	0.913	0.874	0.945	0.865	0.570	0.431
	Human expert	0.954	0.908	0.927	0.862	0.661	0.952	0.909	0.927	0.879	0.660	0.349

AI Artificial intelligence, DWMH Deep and subcortical white matter hyperintensity, MAE Mean absolute error, PVH Periventricular hyperintensity.

In PVH classification, assessed using multi-class accuracy, the AI employing the “Youden-neighbor” method outperformed the human expert on both the Fazekas (AI: 0.798 vs. expert: 0.743) and Brain Dock (AI: 0.706 vs. expert: 0.633) scales. For DWMH, although the human expert achieved higher overall multi-class accuracy, the AI using the “Youden-neighbor“ method demonstrated superior performance at specific boundary classifications: 0/1/2 vs. 3 for Fazekas (AI: 0.954 vs. expert: 0.927) and 0/1/2 vs. 3/4 for Brain Dock (AI: 0.945 vs. expert: 0.927). A similar trend was observed in multi-class F1-scores.

Mean absolute error (MAE) analysis further supported these findings. The AI utilizing the “Youden-neighbor” method achieved lower MAE for PVH on both scales: Fazekas (AI: 0.211 vs. expert: 0.257) and Brain Dock (AI: 0.303 vs. expert: 0.376). For DWMH, the human expert demonstrated lower MAE.

Optimal volume ratio thresholds for Fazekas PVH, determined using the “Youden-neighbor“ method, were 0.00017 (0–1), 0.00402 (1–2), and 0.00977 (2–3); for DWMH, the corresponding thresholds were 0.00013 (0–1), 0.00179 (1–2), and 0.00743 (2–3). Thresholds for alternative methods and Brain Dock scales are provided in Supplementary Table S1.

Figure 1b presents the inter-rater agreement analysis for PVH and DWMH grading. For PVH, the AI’s average agreement with human raters was 0.622 compared with 0.551 among human raters. For DWMH, the AI’s average agreement was 0.630, whereas the human agreement was 0.662.

### Qualitative comparison of grading

**Fig. 2:**
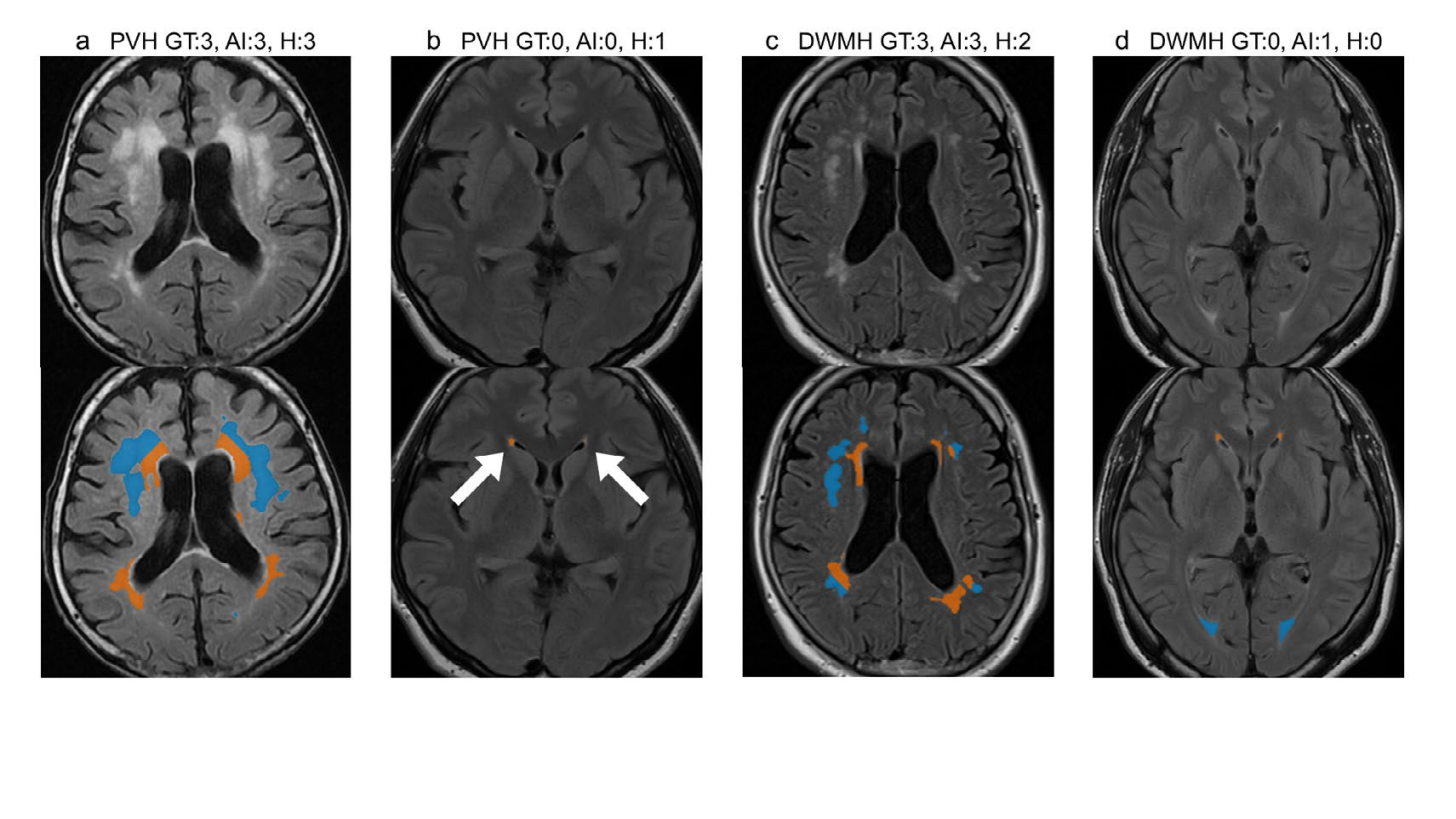
Fazekas scale grading results of representative patients. The top image displays a representative slice from an MRI volume, whereas the bottom image shows AI-predicted PVH (orange) and DWMH (blue) regions for the same slice. Titles indicate grading results (PVH or DWMH) for the entire volume: ground truth grade, AI-predicted grade, and human expert-predicted grade. (**a**) A patient for whom both the AI and the human expert graded correctly. (**b**) A patient for whom only the AI graded PVH correctly. (**c**) A patient for whom only the AI graded DWMH correctly. (**d**) A patient for whom the AI failed in DWMH grading, whereas the human expert’s grade was accurate.

Figure 2 illustrates the Fazekas scale grading results. The top row displays the original slices, whereas the bottom row highlights the AI-predicted PVH (orange) and DWMH (blue) regions, with subtitles indicating the corresponding grades. AI predictions were determined using thresholds derived from the Youden-neighbor method.

Figure 2a presents representative successful cases where both the AI and human experts accurately predicted grades, demonstrating PVH and DWMH separation based on a fixed distance from the lateral ventricles. Figure 2b shows that the AI correctly predicted a PVH grade of 0, whereas the human expert overestimated it as grade 1. The AI detected small PVH regions (indicated by white arrows) but classified them as grade 0, as their volume ratio fell below the threshold. Figure 2c illustrates a case in which the AI accurately predicted a DWMH grade of 3. Figure 2d depicts a case in which the AI failed in DWMH grading, whereas the human expert’s grade was accurate. In this case, the boundary between PVH and DWMH was ambiguous; the AI applied consistent, distance-based rules to make definitive judgments from the ventricular surface. For DWMH grading, although the Fazekas scale provides a qualitative definition, the AI made quantitative decisions based on volume ratios and learned thresholds.

As AI grades are derived from volume ratios, the Youden-neighbor plots exhibit smaller variations in volume ratio distributions within the same grade, with no overlap between neighboring grades (Fig. 1a). Conversely, human-assigned grades—ground truth (GT) and expert assessments—show overlap due to the qualitative nature of grading and subjective judgment. Cases with volume ratios near overlapping regions often reveal discrepancies between the magnitude of the volume ratio and the assigned GT grade. The AI consistently and objectively analyzes these cases (Supplementary Fig. S2).

### Processing time

The average processing speed per volume was 18.5 s, of which approximately 15 s were required for WMH segmentation and 3.5 s for additional tasks, such as lateral ventricle and brain segmentation. The memory usage was approximately 848 mebibytes.

## Discussion

This study developed a novel AI algorithm that automatically calculates the volumes of PVH and DWMH using MRI FLAIR images. The diagnostic performance of the AI was compared with and validated against human readings, with grading based on the internationally recognized Fazekas scale, which was validated against the Japan Brain Dock Society’s original scale^16–18,39,40^. The AI algorithm demonstrated several notable advantages. For PVH grading, the AI achieved higher accuracy compared with the human expert. Although the overall accuracy for DWMH was slightly lower, the AI outperformed human experts in specific boundary cases. Inter-rater agreement analysis indicated that the AI provides consistent grading, exhibiting higher agreement compared with human raters for PVH and comparable agreement for DWMH. These findings suggest that the AI’s quantitative grading approach provides a more stable and objective standard compared with the qualitative assessments used by human raters.

In this study, AI-based grading demonstrated lower performance for DWMH compared with PVH in terms of accuracy, F1-score, MAE, and inter-rater agreement. Several factors may account for this discrepancy. First, the discrepancy may stem from both pathophysiological and definitional differences. PVH is spatially well-defined by its proximity to the ventricles, allowing more consistent interpretation and algorithmic learning. By contrast, DWMH lesions are heterogeneous in shape, location, and signal intensity, often appearing as scattered or confluent foci, which complicates segmentation and classification. Moreover, the Fazekas grade 2 definition—“beginning of confluence”—emphasizes spatial proximity rather than total volume, a feature not fully captured by the volume-based thresholds used in the AI algorithm, potentially leading to boundary misclassifications. When the posterior horn of the lateral ventricle is minimally visible, WMHs in that region are often intuitively interpreted as PVH by human experts. However, as our algorithm strictly classifies WMHs based on distance from the ventricle, these lesions tend to be labeled as DWMH instead (Fig. 2d). Second, as illustrated in Fig. 1a, DWMH volume on the Brain Dock scale increases sharply from grade 2 to grade 3, whereas PVH volume rises more gradually across all grades. This contrast likely reflects differences in the scale definitions. Grade 2 DWMH (“mottled lesions ≥ 3 mm in diameter”) can be satisfied by only a few small lesions, resulting in a relatively low total volume, whereas grade 3 DWMH (“confluent foci in deep white matter”) requires lesion confluence, producing a sudden volumetric surge. By contrast, grade 2 PVH (“extending throughout the periventricular area”) already encompasses the entire ventricular border, and grade 3 PVH (“extending into deep white matter”) represents deeper extension rather than wider spread, yielding more modest volume changes. This abrupt volumetric shift contributes to classification instability, whereby minor discrepancies near the grade boundary may result in misclassification and higher MAE. These findings suggest that a volume-based classification method may be particularly suitable for evaluating DWMH. Third, DWMH is more susceptible to inter-rater variability among expert neuroradiologists compared with PVH, suggesting that the reliability of ground truth labels for DWMH may be inherently lower^41^. This variability can introduce noise during model training, potentially impairing the generalization performance of the AI model and reducing its F1-score.

The AI-based grading approach enabled a clear distinction between PVH and DWMH, providing objective and consistent decisions based on volumetric measurements. Unlike human raters, who rely on qualitative assessments to assign grades, the AI employs a quantitative approach using measured volumes and threshold-based criteria. This quantitative approach likely accounts for its higher consistency, as demonstrated by the inter-rater agreement analysis. Additionally, the AI processes a single volume in approximately 18.5 s on a central processing unit (CPU), supporting its efficiency for clinical application. These features enhance grading reliability and objectivity while significantly reducing physicians’ workloads.

By using both the Fazekas and Brain Dock scales, automatic grading was achieved with accuracy comparable to human performance through the application of volume ratios. Although recent advancements in AI and various algorithms have enabled automated volumetric measurements for WMHs, the accuracy and reliability of these approaches—particularly in separating and grading PVH and DWMH—remain limited, with few clinically applicable solutions reported^1,23,42–44^. This limitation may stem from the age-dependent and heterogeneous nature of PVH and DWMH, which vary in number, shape, and location, thereby presenting challenges for objective evaluation by AI^19,39^.

The key to improving WMH evaluation lies in integrating subjective qualitative assessments from human experts with the quantitative volumetric measurements provided by AI to achieve more objective outcomes. By providing consistent grading, as evidenced by inter-rater agreement analysis, the AI can serve as a stable reference standard to reduce variability in human evaluations and enhance overall grading reliability. Previous studies were limited by small, single-center cohorts of approximately 100 patients and primarily focused on WMHs associated with specific diseases, such as Alzheimer’s disease, cerebral amyloid angiopathy, multiple sclerosis, or hereditary cerebral small vessel disease^45–47^. Most AI studies to date have emphasized WMH segmentation, with little attention devoted to advancing grading techniques^2,21,38,48^. In the future, AI will need to be capable of distinguishing not only the severity of each white matter change but also its underlying etiology by integrating additional clinical and imaging information.

The AI algorithm developed in this study represents a novel risk prediction model capable of distinguishing PVH from DWMH and accurately assessing their severity. To our knowledge, it is the first automatic quantitative grading algorithm for WMHs that reliably differentiates PVH from DWMH, demonstrating clinical feasibility for stroke and dementia risk prediction. By serving as a standardized grading reference, this AI-based algorithm has the potential to improve the accuracy of screening for cognitively healthy individuals at risk of early dementia, facilitating timely lifestyle interventions aimed at preventing or delaying the onset of dementia or stroke.

This study has some limitations. First, it was a retrospective observational study rather than a randomized controlled trial, which introduces the potential for bias. Nevertheless, as a multicenter study, efforts were made to minimize this bias. Second, only FLAIR images were evaluated, which may not adequately differentiate certain conditions, such as lacunar infarctions, where additional imaging sequences are necessary. Plans are underway to develop a next-generation AI algorithm capable of differentiating WMHs from lacunar infarcts by incorporating multiple imaging modalities, such as T1- and T2-weighted imaging. Third, multiple physicians performed the annotations, which could introduce diagnostic bias. However, all participating physicians were board-certified radiologists from the Japan Radiological Society or board-certified neuroradiologists from the Japanese Neurosurgical Society, each with over 10 years of experience, ensuring a high-quality diagnosis. Fourth, the relatively small number of samples included in the final analysis may pose a risk of bias. Although 1,092 patients were recruited from multiple centers, only 246 were analyzed. These patients were selected to balance WMH severity grades based on clinical annotations, but other factors such as age, sex, and disease background were not considered, potentially limiting the generalizability of the findings. Plans are in place to conduct multicenter validation using the developed AI algorithm and expand the sample size in future studies.

In conclusion, the AI algorithm developed in this study effectively distinguishes between PVH and DWMH, achieving accuracy comparable to that of human experts. It provides a reliable and objective reference standard, potentially reducing interobserver variability. These findings underscore the importance of AI-driven quantitative grading as a more consistent alternative to subjective human evaluation.

## Methods

### Ethics approval and informed consent

This study was conducted in accordance with the guidelines of Hiroshima University Hospital and approved by the Ethical Committee for Epidemiology of Hiroshima University (Institutional Review Board of Hiroshima University; approval number: E2022-0262). As the personal data collected during Brain Dock examinations were fully anonymized, the requirement for obtaining individual informed consent was waived by the Ethical Committee for Epidemiology of Hiroshima University. As the data were anonymized from the outset, the authors had no access to any personally identifiable participant information during data collection.

### Study design

#### Dataset

This study utilized 1,092 MRI FLAIR images collected from three Japanese hospitals^1^. Due to the costs associated with grading annotations, 246 images were randomly selected to ensure a uniform distribution of WMH severity. During this selection, other factors such as age, sex, and disease background were not considered.

Of these 246 images, 207 had been used in our previous WMH segmentation study^1^. Among them, training images were reused exclusively for threshold learning, while the 69 evaluation images were again used solely for performance assessment in this study. To further balance the grade distribution in the evaluation set, 40 additional cases (20 from grade 0 and 20 from grade 4) were randomly selected from previously unannotated images in our earlier dataset.

In total, 137 images were allocated to the training set and 109 to the test set. The training set included 85 men and 52 women, with a mean age of 65.2 years (SD = 10.7; range: 41–85). The test set comprised 51 men and 58 women, with a mean age of 67.9 years (SD = 11.1; range: 34–88). Slice thickness ranged from 5 to 6 mm.

#### WMH grading scales

The Fazekas scale, an internationally recognized standard, evaluates PVH and DWMH on a 4-point scale. The Brain Dock scale similarly assesses PVH and DWMH but employs a 5-point system, dividing grade 3 of the Fazekas scale into two separate grades. In this study, annotations were performed according to the Brain Dock scale, with grades 3 and 4 merged during validation to align with the Fazekas scale (Supplementary Fig. S3).

#### Comparison of grading performance

For the 109 test images, WMHs were graded by three neuroradiologists who established the GT and an additional rater. The GT was determined by consensus among the three neuroradiologists, with any disagreements resolved by majority vote. In cases where all three radiologists had assigned different grades—a theoretical possibility due to the multi-class nature of the task—consensus would have been achieved through discussion. However, no such cases occurred in this study. The grading of the fourth raters served as the representative human expert value for comparison with AI performance. For the 137 training images, WMHs were graded in a single round by four different neuroradiologists who were not involved in GT creation or AI evaluation. Grading performance was assessed by comparing the agreement between the human expert and AI with the GT using standard classification metrics.

### WMH grading algorithm

#### Overview of WMH grading algorithm

**Fig. 3:**
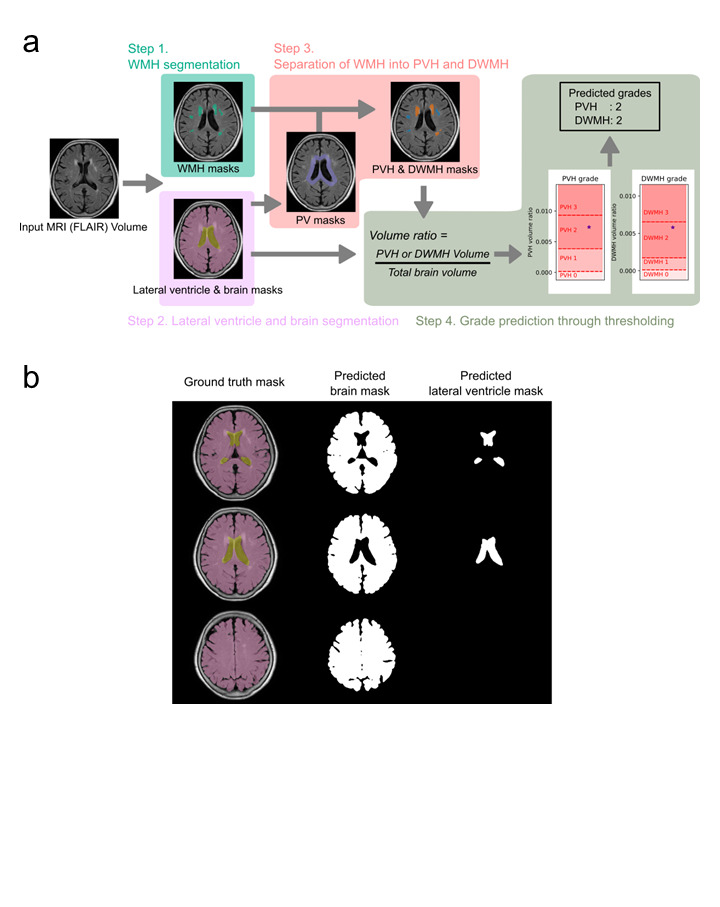
(**a**) Flow of the proposed WMH grading algorithm. The algorithm comprises four steps: (1) WMH segmentation, (2) lateral ventricle and brain segmentation, (3) separation of WMH into PVH and DWMH, and (4) grade prediction through thresholding. Each step is depicted with a distinct background color, accompanied by illustrative images and text to demonstrate the workflow. (**b**) Segmentation examples of the lateral ventricles and brain. The left column shows the ground truth masks, with the brain depicted in pink and the lateral ventricles in yellow, generated using SynthSeg. The middle and right columns display the segmentation predictions produced by the U-Net model for the lateral ventricles and brain, as implemented in the proposed method.

The proposed method for automatic WMH grading (Fig. 3a) consists of four steps: (1) WMH segmentation, (2) segmentation of the lateral ventricles and brain, (3) separation of WMH into PVH and DWMH, and (4) grade prediction via thresholding. FLAIR images were used as the sole input throughout this process. In this study, FLAIR images had a slice thickness of 5–6 mm. No spatial resampling along the axial direction was performed, as the algorithm was designed to accommodate variations in native slice thickness without interpolation.

#### WMH segmentation

WMH regions on FLAIR images were segmented using a U-Net–based ensemble model comprising two variants: one with an EfficientNet-B5 backbone and the other with a ResNext50 backbone. The models were implemented in PyTorch and trained using the Adam optimizer (initial learning rate = 0.001, batch size = 15, epochs = 15) with Matthews correlation coefficient loss and a cosine-annealing learning-rate schedule. Details of the model architecture, training protocol, and evaluation were specified based on our prior publication^1^. FLAIR images were used as the sole input to ensure high processing efficiency and compatibility with thick-slice clinical MRI^1^.

### Lateral ventricles and brain segmentation

PVH and DWMH classifications and WMH volume normalization relied on a U-Net model with a ResNet18 backbone to segment the lateral ventricles and brain parenchyma. The model, trained on a dataset from a previous study^1^, achieved a Dice score of 0.958 when compared with GT masks. These GT masks, representing the brain parenchyma and lateral ventricle masks, were initially generated using SynthSeg^49^, a deep learning tool for brain segmentation across various contrasts and resolutions, and were subsequently manually refined (Fig. 3b). SynthSeg was not used in the final workflow due to redundancy and its higher computational cost.

### Separation of PVH and DWMH

Methods for separating PVH and DWMH vary across studies. Griffanti et al. reported that the 10-mm distance rule— defining a 10-mm boundary from the ventricular surface as the decision criterion—provided optimal separation for the tested factors^50^. This threshold has been widely adopted in previous neuroimaging studies as a practical and reproducible anatomical criterion for distinguishing PVH from DWMH^51,52^. Moreover, the cutoff was originally validated in a large cohort of older adults, demonstrating robust associations with cognitive performance, tissue microstructure, and cardiovascular risk factors^49^. In this study, this empirically supported threshold was adopted without additional histopathological calibration, and an algorithm was developed to automate this rule.

A PV mask was generated within 10 mm of the lateral ventricles, and WMH regions were classified on each axial slice. Regions within the PVH mask were classified as PVH, those outside as DWMH, and boundary-spanning regions as PVH if more than 60% fell within the PV mask. Otherwise, the regions were split into PVH and DWMH.

The 60% threshold was determined based on an ablation study during algorithm development. Multiple candidate thresholds (50%, 70%, 80%, and 90%) were evaluated and compared for multiclass grading accuracy on the training set (Supplementary Table S2). This threshold yielded the highest accuracy and was therefore adopted. A similar rule was not applied to DWMH, as boundary-spanning lesions were relatively rare and had limited impact on grading. Moreover, simplicity and interpretability of the algorithm were prioritized.

To reduce computational cost, the PV mask was approximated using image processing techniques. The lateral ventricle mask was expanded by 10 mm via two-dimensional and three-dimensional morphological dilation based on pixel and slice spacing. The final PV mask was obtained by subtracting the original ventricle region from the union of the expanded areas.

### Grading method using thresholds

Grade prediction was performed by applying thresholds to PVH and DWMH volumes normalized by total brain volume, referred to as volume ratios, to account for inter-individual differences in brain size. Total brain volume was calculated using the brain masks generated by the lateral ventricles and brain segmentation model. Optimal volume ratio thresholds were determined based on the distribution of volume ratios and their corresponding grades in the training dataset.

Two methods were compared for threshold determination. The first, probability density distribution, estimated the volume ratio density for each grade using kernel density estimation and set thresholds at the midpoints between adjacent peaks. The second, Youden’s index maximization, treated grading as a binary classification problem and employed receiver operating characteristic analysis to identify thresholds that maximize the Youden’s index (sensitivity + specificity − 1). This method included two strategies: Youden-all, which analyzed all data at each boundary by grouping multiple classes (e.g., 0/1 vs. 2/3), and Youden-neighbor, which only considered adjacent classes (e.g., 0 vs. 1 and 1 vs. 2). These strategies were used solely for determining the optimal threshold. Importantly, they were independent of the evaluation classification procedure, which assesses all data at each boundary by grouping multiple classes.

All threshold learning procedures were implemented in Python, with kernel density estimation performed using scikit-learn’s KernelDensity module and Youden index maximization using scikit-learn’s ROC analysis tools.

### Evaluation metrics

Grading performance was evaluated using binary classification accuracy at grade boundaries, following previous studies^18^. For the Fazekas scale, PVH and DWMH classifications included 0 vs. 1/2/3, 0/1 vs. 2/3, and 0/1/2 vs. 3. For the Brain Dock scale, the classifications included 0 vs. 1/2/3/4, 0/1 vs. 2/3/4, 0/1/2 vs. 3/4, and 0/1/2/3 vs. 4. This approach reflects clinical WMH grading by assessing whether a grade falls below or above a diagnostic threshold. Multi-class evaluations were also performed for the 4-grade Fazekas scale and the 5-grade Brain Dock scale. Performance metrics included accuracy, F1-score, and MAE to quantify prediction errors.

Inter-rater agreement was evaluated using Fleiss’ kappa to measure the consistency among the four raters who graded the test images, and Cohen’s kappa was used to assess the agreement between the AI and each rater. This analysis was conducted to compare the AI’s performance against human variability and evaluate its potential to provide consistent grading standards.

Processing time was assessed in a CPU environment suitable for clinical settings without high-performance graphics processing units. Measurements were conducted on an Intel Core i5-10500T CPU at 2.30 GHz with 16 gigabytes of memory.

## Supplementary Information

Below is the link to the electronic supplementary material.

Supplementary Material 1

Supplementary Material 2

Supplementary Material 3

Supplementary Material 4

Supplementary Material 5

## Data Availability

The anonymized data from this study are available from the corresponding author upon reasonable request, provided that the requester is a qualified researcher and obtains approval from the institutional review board.
