# Supplementary material for: Advantage of grading classification using volumetric artificial intelligence for periventricular hyperintensity and deep subcortical white matter hyperintensity
Source: Sci Rep. 2025 Nov 17;15:40186. doi: 10.1038/s41598-025-23859-2 (PMC12624063; doi:10.1038/s41598-025-23859-2)
Supplement: Supplementary file 1 — Supplementary Material 1 [file 41598_2025_23859_MOESM1_ESM.pdf]

a

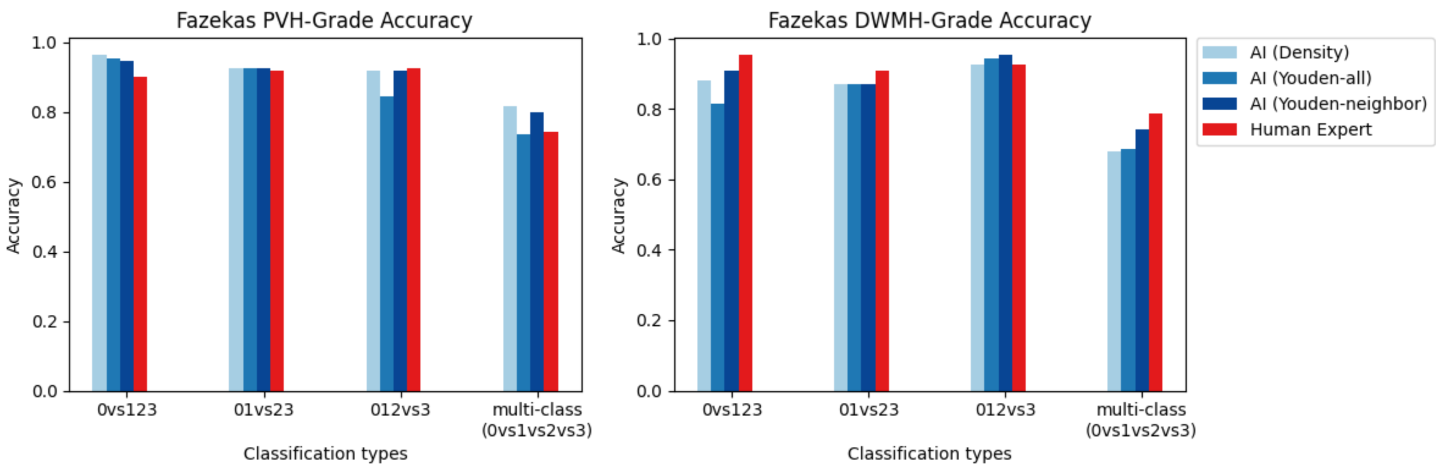

b

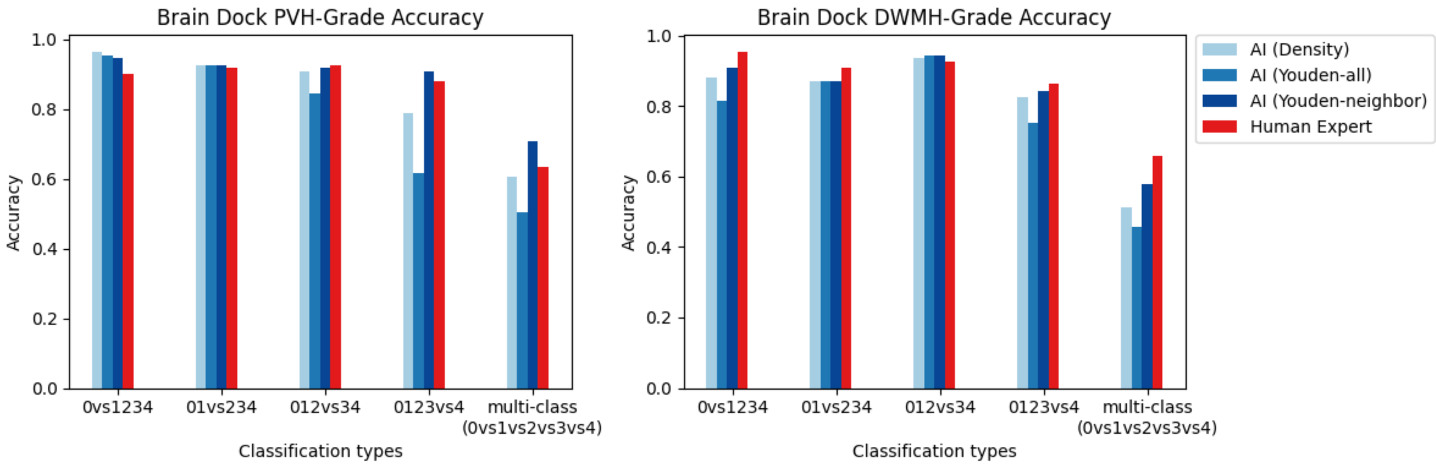

Supplementary Fig. S1. Grading accuracy of the proposed AI method and the human expert

This figure compares the grading accuracy of the AI (shades of blue) with that of the human expert (red).

a. Fazekas scale grading accuracy. The left plot represents accuracy for PVH, whereas the right plot represents accuracy for DWMH.

The horizontal axis shows the evaluation methods, including binary classification (0 vs. 123, 01 vs. 23, 012 vs. 3) and multi-class classification.

b. Brain Dock scale grading accuracy. Similarly, the left plot shows PVH accuracy, whereas the right plot shows DWMH accuracy.

The horizontal axis displays the evaluation methods, including binary classification (0 vs. 1234, 01 vs. 234, 012 vs. 34, and 0123 vs. 4) and multi-class classification.
