# Supplementary material for: Advantage of grading classification using volumetric artificial intelligence for periventricular hyperintensity and deep subcortical white matter hyperintensity
Source: Sci Rep. 2025 Nov 17;15:40186. doi: 10.1038/s41598-025-23859-2 (PMC12624063; doi:10.1038/s41598-025-23859-2)
Supplement: Supplementary file 2 — Supplementary Material 2 [file 41598_2025_23859_MOESM2_ESM.pdf]

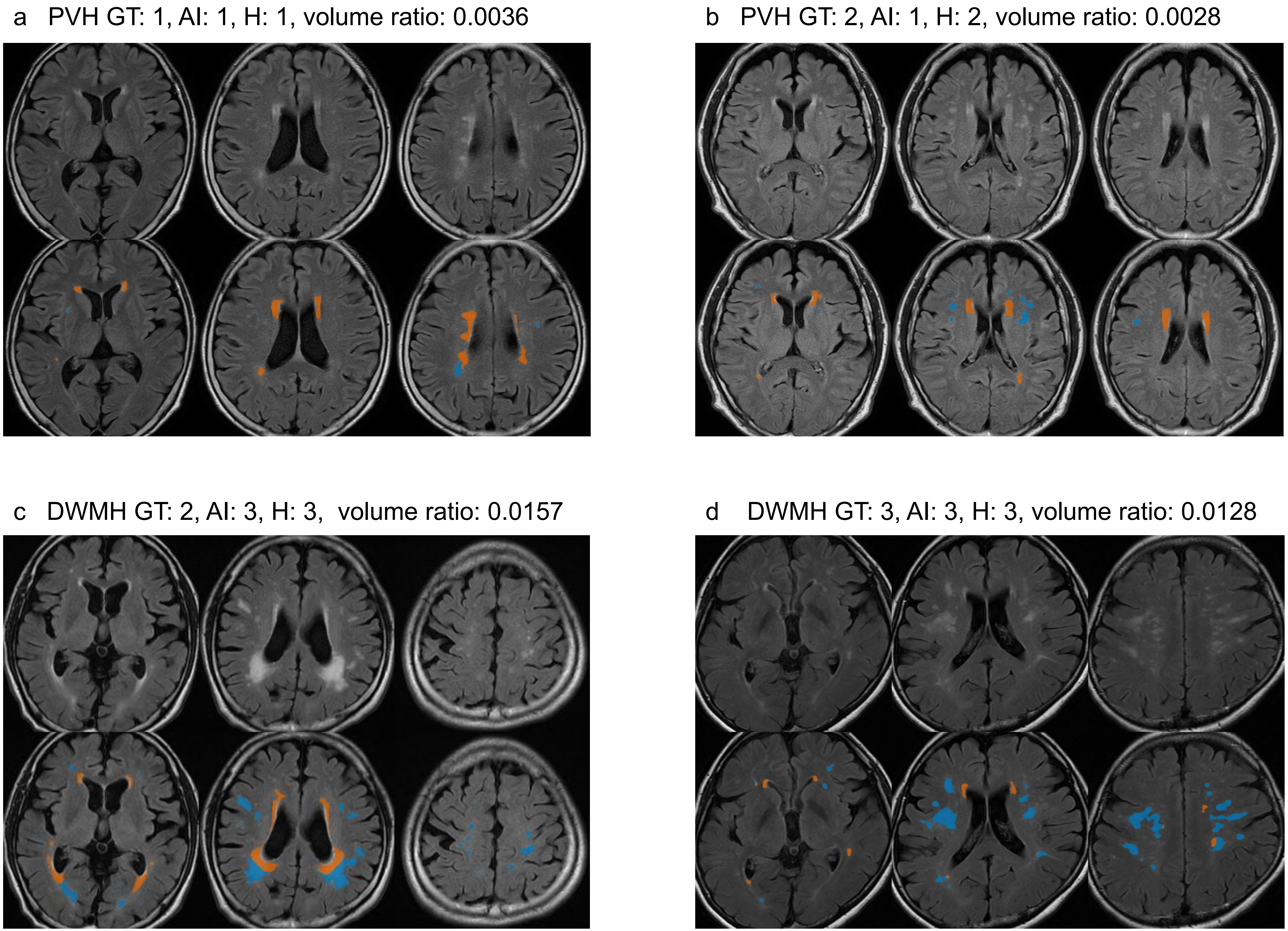

Supplementary Fig. S2. Representative patients illustrating mismatch between GT grades and volume ratios

The top row displays three representative slices from an MRI volume, whereas the bottom row shows AI-predicted PVH (orange) and DWMH (blue) regions for the corresponding slices.

Titles indicate grading results (PVH or DWMH) for the entire volume, including ground truth grade, AI-predicted grade, human expert-predicted grade, and volume ratio.

a, b. Patients in whom PVH GT grades (1 < 2) are inconsistent with the volume ratios (0.0036 > 0.0028). Each patient includes three slices per volume along with their respective PVH volume ratios.

c, d. Patients in whom DWMH GT grades (2 < 3) are inconsistent with the volume ratios (0.0157 > 0.0128). Each patient includes three slices per volume along with their respective DWMH volume ratios.
