# Supplementary material for: Advantage of grading classification using volumetric artificial intelligence for periventricular hyperintensity and deep subcortical white matter hyperintensity
Source: Sci Rep. 2025 Nov 17;15:40186. doi: 10.1038/s41598-025-23859-2 (PMC12624063; doi:10.1038/s41598-025-23859-2)
Supplement: Supplementary file 3 — Supplementary Material 3 [file 41598_2025_23859_MOESM3_ESM.pdf]

a

	Our research's (Brain Dock) PVH scale		Fazekas PVH scale
Grade 0	Absence	Grade 0	Absence
Grade I	Periventricular cap or periventricular rim	Grade I	"Cap" or pencil-thin lining
Grade II	Extending throughout whole periventricular area	Grade II	Smooth "halo"
Grade III	Extending into deep white matter	Grade III	Irregular PVH extending into the deep white matter
Grade IV	Widely extending into the subcortical white matter		

b

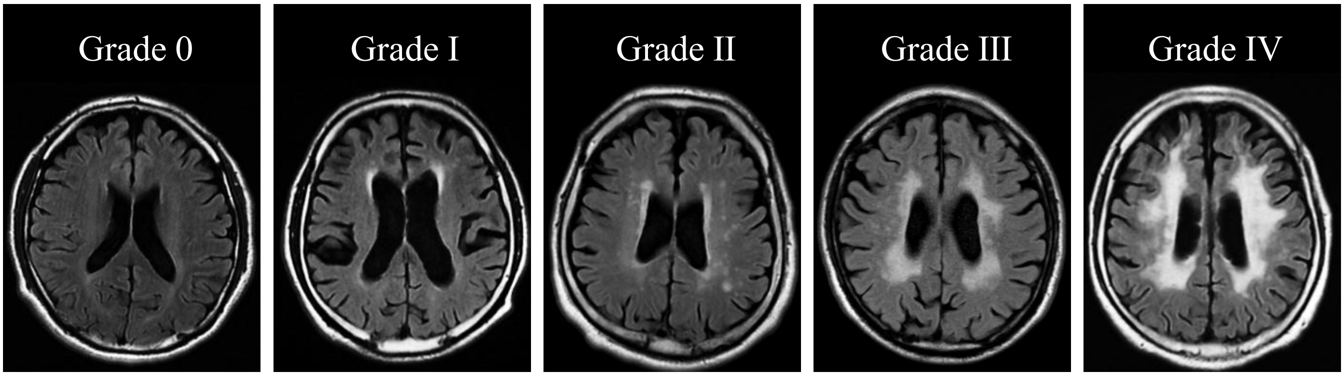

c

	Our research's (Brain Dock) DWMH scale		Fazekas DWMH scale
Grade 0	Absence	Grade 0	Absence
Grade 1	Spotted lesions <3 mm in diameter	Grade 1	Punctate foci
Grade 2	Mottled lesions ≥3 mm in diameter	Grade 2	Beginning confluence of foci
Grade 3	Confluent foci on deep white matter	Grade 3	Large confluent areas
Grade 4	Confluence widely distributed across most of white matter		

d

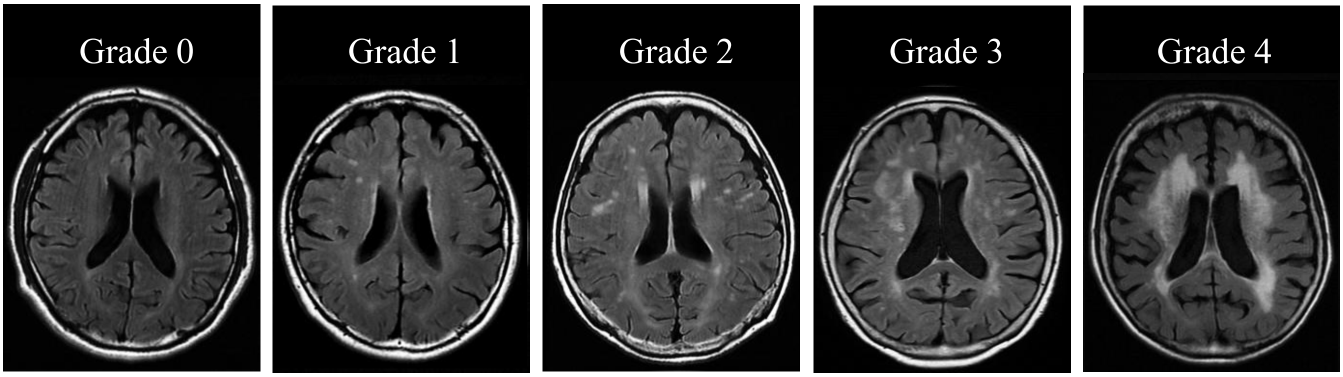

Supplementary Fig. S3. Brain Dock PVH and DWMH scales

a. Definitions of the Brain Dock PVH scale are presented alongside the corresponding Fazekas PVH scale definitions

b. Representative slices illustrating sample cases for each grade of the Brain Dock PVH scale are provided to illustrate the grading criteria.

c. Definitions of the Brain Dock DWMH scale are presented alongside the corresponding Fazekas DWMH scale definitions.

d. Representative slices of sample cases for each grade of the Brain Dock DWMH scale are shown, demonstrating the grading approach.
