# Supplementary material for: Advantage of grading classification using volumetric artificial intelligence for periventricular hyperintensity and deep subcortical white matter hyperintensity
Source: Sci Rep. 2025 Nov 17;15:40186. doi: 10.1038/s41598-025-23859-2 (PMC12624063; doi:10.1038/s41598-025-23859-2)
Supplement: Supplementary file 4 — Supplementary Material 4 [file 41598_2025_23859_MOESM4_ESM.pdf]

Supplementary Table S1. Optimal grading thresholds

	Method	0vs123	01vs23	012vs3	
Fazekas PVH	Density	0.00052	0.00385	0.01012	
	Youden-all	0.00041	0.00402	0.00646	
	Youden-neighbor	0.00017	0.00402	0.00977	
Fazekas DSWMH	Density	0.00027	0.00193	0.01185	
	Youden-all	0.00065	0.00179	0.00687	
	Youden-neighbor	0.00013	0.00179	0.00743	
		0vs1234	01vs234	012vs34	0123vs4
Brain Dock PVH	Density	0.00052	0.00385	0.00933	0.01473
	Youden-all	0.00041	0.00402	0.00646	0.00495
	Youden-neighbor	0.00017	0.00402	0.01008	0.02394
Brain Dock DSWMH	Density	0.00027	0.00193	0.00927	0.02169
	Youden-all	0.00065	0.00179	0.00687	0.01413
	Youden-neighbor	0.00013	0.00179	0.00687	0.02393

DWMH: deep and subcortical white matter hyperintensity, PVH: periventricular hyperintensity
