# Supplementary material for: Advantage of grading classification using volumetric artificial intelligence for periventricular hyperintensity and deep subcortical white matter hyperintensity
Source: Sci Rep. 2025 Nov 17;15:40186. doi: 10.1038/s41598-025-23859-2 (PMC12624063; doi:10.1038/s41598-025-23859-2)
Supplement: Supplementary file 5 — Supplementary Material 5 [file 41598_2025_23859_MOESM5_ESM.pdf]

Supplementary Table S2. Ablation study of boundary classification thresholds for PVH assignment

Threshold	Accuracy (multi-class)	
	PVH	DWMH
50%	0.752	0.645
60%	0.752	0.667
70%	0.752	0.659
80%	0.745	0.652
90%	0.737	0.659

The threshold represents the minimum proportion of a boundary-spanning lesion within the PV mask required for PVH classification. The 60% cutoff yielded superior multiclass accuracy.
PVH: periventricular hyperintensity
